# Supplementary material for: Allosteric regulation and crystallographic fragment screening of SARS-CoV-2 NSP15 endoribonuclease
Source: Nucleic Acids Res. 2023 Apr 28;51(10):5255–70. doi: 10.1093/nar/gkad314 (PMC10250223; doi:10.1093/nar/gkad314)
Supplement: gkad314_Supplemental_Files [file gkad314_supplemental_files.zip › Supplementary datasets.zip!/160/Refine_8/01-BUSTER/Cycle-3/shell.01/R_Rfree.html]

# Rxpct factors in resolution bins at cycle 0


|  |  |  |  |  |  |  |  |  |
| --- | --- | --- | --- | --- | --- | --- | --- | --- |
|  | **<d> (Å)**  Rxpct\_Work | Rxpct\_Free | Rxpct\_All | |  |  |  |  |
|  | 14.6046 | 0.3854 | 0.5754 | 0.3921 |  |
|  | 8.4320 | 0.2481 | 0.2759 | 0.2498 |  |
|  | 6.5314 | 0.2173 | 0.2639 | 0.2199 |  |
|  | 5.5200 | 0.1865 | 0.2422 | 0.1887 |  |
|  | 4.8682 | 0.1507 | 0.2049 | 0.1537 |  |
|  | 4.4035 | 0.1516 | 0.1857 | 0.1535 |  |
|  | 4.0506 | 0.1558 | 0.1853 | 0.1571 |  |
|  | 3.7709 | 0.1619 | 0.2402 | 0.1660 |  |
|  | 3.5421 | 0.1670 | 0.2016 | 0.1688 |  |
|  | 3.3505 | 0.1746 | 0.2432 | 0.1784 |  |
|  | 3.1870 | 0.1797 | 0.2362 | 0.1828 |  |
|  | 3.0453 | 0.1822 | 0.2304 | 0.1843 |  |
|  | 2.9209 | 0.1926 | 0.2136 | 0.1935 |  |
|  | 2.8107 | 0.1878 | 0.2445 | 0.1904 |  |
|  | 2.7120 | 0.1914 | 0.2190 | 0.1929 |  |
|  | 2.6231 | 0.1985 | 0.2175 | 0.1996 |  |
|  | 2.5423 | 0.2103 | 0.2196 | 0.2109 |  |
|  | 2.4686 | 0.2108 | 0.2244 | 0.2115 |  |
|  | 2.4010 | 0.2225 | 0.2330 | 0.2230 |  |
|  | 2.3386 | 0.2524 | 0.2700 | 0.2532 |  |
|  | **Overall** | 0.1901 | 0.2346 | 0.1924 |  |

# Rxpct factors in resolution bins at cycle 1


|  |  |  |  |  |  |  |  |  |
| --- | --- | --- | --- | --- | --- | --- | --- | --- |
|  | **<d> (Å)**  Rxpct\_Work | Rxpct\_Free | Rxpct\_All | |  |  |  |  |
|  | 14.6046 | 0.3886 | 0.5800 | 0.3953 |  |
|  | 8.4320 | 0.2499 | 0.2770 | 0.2515 |  |
|  | 6.5314 | 0.2175 | 0.2653 | 0.2201 |  |
|  | 5.5200 | 0.1859 | 0.2413 | 0.1881 |  |
|  | 4.8682 | 0.1499 | 0.2051 | 0.1529 |  |
|  | 4.4035 | 0.1510 | 0.1854 | 0.1529 |  |
|  | 4.0506 | 0.1550 | 0.1848 | 0.1563 |  |
|  | 3.7709 | 0.1614 | 0.2408 | 0.1656 |  |
|  | 3.5421 | 0.1669 | 0.2023 | 0.1687 |  |
|  | 3.3505 | 0.1746 | 0.2453 | 0.1785 |  |
|  | 3.1870 | 0.1801 | 0.2365 | 0.1833 |  |
|  | 3.0453 | 0.1827 | 0.2308 | 0.1849 |  |
|  | 2.9209 | 0.1929 | 0.2146 | 0.1939 |  |
|  | 2.8107 | 0.1879 | 0.2433 | 0.1905 |  |
|  | 2.7120 | 0.1910 | 0.2190 | 0.1924 |  |
|  | 2.6231 | 0.1985 | 0.2177 | 0.1997 |  |
|  | 2.5423 | 0.2100 | 0.2209 | 0.2106 |  |
|  | 2.4686 | 0.2102 | 0.2230 | 0.2108 |  |
|  | 2.4010 | 0.2223 | 0.2341 | 0.2229 |  |
|  | 2.3386 | 0.2521 | 0.2702 | 0.2529 |  |
|  | **Overall** | 0.1901 | 0.2351 | 0.1924 |  |

# Rxpct factors in resolution bins at cycle 2


|  |  |  |  |  |  |  |  |  |
| --- | --- | --- | --- | --- | --- | --- | --- | --- |
|  | **<d> (Å)**  Rxpct\_Work | Rxpct\_Free | Rxpct\_All | |  |  |  |  |
|  | 14.6046 | 0.3898 | 0.5816 | 0.3965 |  |
|  | 8.4320 | 0.2505 | 0.2776 | 0.2520 |  |
|  | 6.5314 | 0.2171 | 0.2647 | 0.2197 |  |
|  | 5.5200 | 0.1856 | 0.2409 | 0.1879 |  |
|  | 4.8682 | 0.1493 | 0.2054 | 0.1525 |  |
|  | 4.4035 | 0.1507 | 0.1853 | 0.1527 |  |
|  | 4.0506 | 0.1547 | 0.1846 | 0.1560 |  |
|  | 3.7709 | 0.1614 | 0.2405 | 0.1656 |  |
|  | 3.5421 | 0.1671 | 0.2028 | 0.1689 |  |
|  | 3.3505 | 0.1749 | 0.2439 | 0.1786 |  |
|  | 3.1870 | 0.1798 | 0.2364 | 0.1830 |  |
|  | 3.0453 | 0.1826 | 0.2309 | 0.1848 |  |
|  | 2.9209 | 0.1928 | 0.2126 | 0.1937 |  |
|  | 2.8107 | 0.1879 | 0.2431 | 0.1904 |  |
|  | 2.7120 | 0.1909 | 0.2183 | 0.1923 |  |
|  | 2.6231 | 0.1983 | 0.2169 | 0.1994 |  |
|  | 2.5423 | 0.2098 | 0.2202 | 0.2104 |  |
|  | 2.4686 | 0.2100 | 0.2228 | 0.2107 |  |
|  | 2.4010 | 0.2220 | 0.2337 | 0.2225 |  |
|  | 2.3386 | 0.2519 | 0.2700 | 0.2528 |  |
|  | **Overall** | 0.1901 | 0.2349 | 0.1923 |  |

# Rxpct factors in resolution bins at cycle 3


|  |  |  |  |  |  |  |  |  |
| --- | --- | --- | --- | --- | --- | --- | --- | --- |
|  | **<d> (Å)**  Rxpct\_Work | Rxpct\_Free | Rxpct\_All | |  |  |  |  |
|  | 14.6046 | 0.3889 | 0.5845 | 0.3957 |  |
|  | 8.4320 | 0.2474 | 0.2760 | 0.2491 |  |
|  | 6.5314 | 0.2126 | 0.2595 | 0.2152 |  |
|  | 5.5200 | 0.1814 | 0.2357 | 0.1836 |  |
|  | 4.8682 | 0.1473 | 0.2041 | 0.1505 |  |
|  | 4.4035 | 0.1489 | 0.1858 | 0.1510 |  |
|  | 4.0506 | 0.1534 | 0.1849 | 0.1548 |  |
|  | 3.7709 | 0.1608 | 0.2410 | 0.1650 |  |
|  | 3.5421 | 0.1671 | 0.2035 | 0.1690 |  |
|  | 3.3505 | 0.1748 | 0.2450 | 0.1787 |  |
|  | 3.1870 | 0.1797 | 0.2359 | 0.1829 |  |
|  | 3.0453 | 0.1828 | 0.2274 | 0.1848 |  |
|  | 2.9209 | 0.1929 | 0.2130 | 0.1938 |  |
|  | 2.8107 | 0.1881 | 0.2398 | 0.1905 |  |
|  | 2.7120 | 0.1900 | 0.2197 | 0.1915 |  |
|  | 2.6231 | 0.1984 | 0.2183 | 0.1996 |  |
|  | 2.5423 | 0.2103 | 0.2210 | 0.2109 |  |
|  | 2.4686 | 0.2113 | 0.2230 | 0.2119 |  |
|  | 2.4010 | 0.2232 | 0.2325 | 0.2236 |  |
|  | 2.3386 | 0.2520 | 0.2680 | 0.2527 |  |
|  | **Overall** | 0.1890 | 0.2343 | 0.1912 |  |
